# 1α,11α,15β-Triacet­oxy-7β-hydroxy-7α,20-ep­oxy-*ent*-kaur-16-en-6-one

**DOI:** 10.1107/S1600536809041828

**Published:** 2009-10-17

**Authors:** Fu-Lin Yan, Xue-Mei Di, Chuang Feng, Rei-Jie Hou

**Affiliations:** a School of Pharmacy, Xinxiang Medical University, Xinxiang, Henan 453003, People’s Republic of China

## Abstract

The title compound, C_26_H_34_O_9_, a natural *ent*-kaurane diterpenoid, is composed of four rings with the expected *cis* and *trans* junctions. In the crystal structure, the mol­ecules stack along the *a* axis and are linked together by inter­molecular O—H⋯O hydrogen bonds.

## Related literature

For the genus Isodon and diterpenoids, see: Sun *et al.* (2001 ▶); Li *et al.* (2006 ▶); Yan *et al.* (2009 ▶). For hydorgen bonds, see: Nardelli (1995 ▶). For a description of the Cambridge Structural Database, see: Allen *et al.* (1987 ▶).
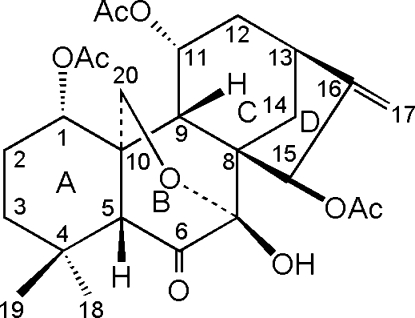


## Experimental

### 

#### Crystal data

C_26_H_34_O_9_
*M* *_r_* = 490.53
Orthorhombic, 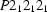

*a* = 11.3317 (5) Å
*b* = 11.5061 (4) Å
*c* = 19.0663 (6) Å
*V* = 2485.93 (16) Å^3^
*Z* = 4
Mo *K*α radiation
μ = 0.10 mm^−1^
*T* = 93 K
0.43 × 0.37 × 0.33 mm

#### Data collection

Rigaku AFC10/Saturn724+ diffractometer
Absorption correction: none
16790 measured reflections
3165 independent reflections
3073 reflections with *I* > 2σ(*I*)
*R*_int_ = 0.029

#### Refinement

*R*[*F* ^2^ > 2σ(*F* ^2^)] = 0.034
*wR*(*F* ^2^) = 0.085
*S* = 1.00
3165 reflections
325 parameters
H atoms treated by a mixture of independent and constrained refinement
Δρ_max_ = 0.21 e Å^−3^
Δρ_min_ = −0.18 e Å^−3^

### 

Data collection: *CrystalClear* (Rigaku, 2008 ▶); cell refinement: *CrystalClear*; data reduction: *CrystalClear*; program(s) used to solve structure: *SHELXS97* (Sheldrick, 2008 ▶); program(s) used to refine structure: *SHELXL97* (Sheldrick, 2008 ▶); molecular graphics: *SHELXTL* (Sheldrick, 2008 ▶); software used to prepare material for publication: *SHELXTL*.

## Supplementary Material

Crystal structure: contains datablocks global, I. DOI: 10.1107/S1600536809041828/jj2009sup1.cif

Structure factors: contains datablocks I. DOI: 10.1107/S1600536809041828/jj2009Isup2.hkl

Additional supplementary materials:  crystallographic information; 3D view; checkCIF report

## Figures and Tables

**Table 1:** Hydrogen-bond geometry (Å, °)

*D*—H⋯*A*	*D*—H	H⋯*A*	*D*⋯*A*	*D*—H⋯*A*
O5—H5*O*⋯O3^i^	0.89 (3)	2.18 (3)	2.853 (2)	133 (3)

Symmetry code: (i) 
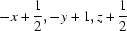
.

**Fig. 1.**
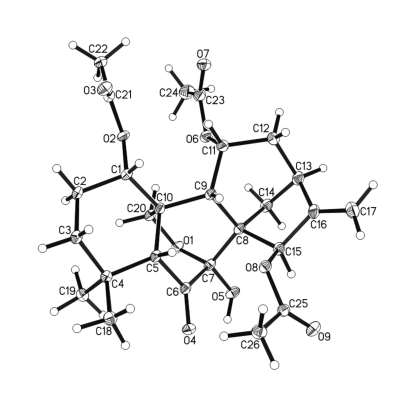
A view of the molecular structure of compound (I). Displacement ellipsoids are drawn at the 50% probability level.

**Fig. 2.**
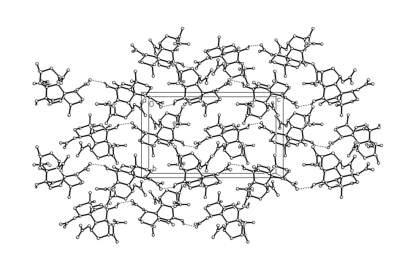
The crystal packing of (I), viewed along the a axis, showing the O–H···O hydrogen bonds as dashed lines.

## supplementary crystallographic information

### Comment

The title compound, (I), C_26_H_34_O_9_, is a natural *ent*-kaurane diterpenoid isolated from the medicinal plant Isodon Japonica. The leaves of this plant have been used as an antibacterial, anti-inflammatory and stomachic agent.  The structure of compound (I), derived from Isodon Parvifolius, has been postulated from spectroscopic methods (Li *et al.*, 2006). As a conformation of (I) the crystal structure analysis has been determined here and confirms the structure proposed above.  One hydroxyl group adopts a β-orientation at C7, and three acetoxyl groups adopt α,α,β-orientations at C1,C11 and C15, respectively, Fig.1. A *trans* junction occurs between ring A (C1—C5/C10) and ring B (C5—C10) while *cis* junctions are present between rings B and C (C8/C9/C11—C14), and between rings C and D (C8/C13—C16).  Bond lengths and angles are within expected ranges (Allen *et al.*, 1987), with average values (Å): C*sp*^3^—C*sp*^3^ = 1.542 (3), C*sp*^3^—C*sp*^2^ = 1.509 (3), C*sp*^2^—C*sp*^2^ (CC) = 1.320 (3), CO = 1.207 (2), C*sp*^3^—O = 1.438 (2), and C*sp*^2^—O = 1.351 (2). Ring A adopts a chair conformation, with an average torsion angle of 51.5 (2) °. Rings B and C adopt a boat conformation because of the formation of an oxygen bridge at C-7 and C-20. Ring D shows an envelope conformation; the flap atom, C14, lies 0.7648 (0.0028) Å from the plane defined by atoms C8, C15, C16 and C13. In addition, the six-membered rings O1/C20/C10/C5—C7 and O1/C7—C10/C20 both adopt boat conformations. Compound (I) contains nine chiral centers at C1(S), C5(*R*), C7(S), C8(S), C9(S), C10(S), C11(*R*), C13(S) and C15(*R*).  Although the absolute configuration could not be reliably determined from anomalous dispersion effects, the negative optical rotation showed this compound to be in the *ent*-kaurane series as reported in genus Isodon (Sun *et al.*,2001), rather than in the Kaurane series, which allowed us to assign the correct configuration.  The title molecule is characterized by the formation of O–H···O hydrogen-bonds (Table 1, Nardelli, 1995).  The strong hydrogen bond O–H···O interaction is responsible for crystal growth in [100] direction, Fig. 2. Indeed, in the substructure, atom O5 in the molecule at (x, y, z) acts as a hydrogen bond donor to the carbonyl O3 atom in the molecule at (-x +1/2, -y +1, z +1/2).

### Experimental

The dried and crushed leaves of Isodon Japonica (17 kg, collected from Tongbai Prefecture, Henan Province, China) were extracted four times with Me_2_CO/H_2_O (7:3, *v*/*v*) at room temperature over a period of six days. The extract was filtered and the solvent was removed under reduced pressure. The residue was then partitioned between water and AcOEt. After removal of the solvent, the AcOEt residue was separated by repeated silica gel (200–300 mesh) column chromatography and recrystallization from CHCl_3_/Me_2_CO(10:1), giving 60 mg of compound (I) (m.p. 430–432 K. Optical rotation: [α]_D_^22^ -74.6 ° (c 0.45, MeOH). Crystals suitable for X-ray analysis were obtained by slow evaporation of a solution of compound (I) in Me_2_CO at room temperature.

### Refinement

All H atoms were included in calculated positions and refined as riding atoms, with C—H = 0.98Å (CH_3_), 0.99Å (CH_2_), and 1.00Å (CH), and with *U*_iso_(H) = 1.2 *U*_eq_(C). In the absence of significant anomalous scattering effects, Friedel pairs were merged. The choice of enantiomer was based on comparison of the optical rotation with that of related compounds with known stereochemistry.

### Crystal data

**Table:**

C_26_H_34_O_9_	*F*(000) = 1048
*M**_r_* = 490.53	*D*_x_ = 1.311 Mg m^−^^3^
Orthorhombic, *P*2_1_2_1_2_1_	Mo *K*α radiation, λ = 0.71073 Å
Hall symbol: P 2ac 2ab	Cell parameters from 8353 reflections
*a* = 11.3317 (5) Å	θ = 3.2–27.5°
*b* = 11.5061 (4) Å	µ = 0.10 mm^−^^1^
*c* = 19.0663 (6) Å	*T* = 93 K
*V* = 2485.93 (16) Å^3^	Block, colorless
*Z* = 4	0.43 × 0.37 × 0.33 mm

### Data collection

**Table:**

Rigaku AFC10/Saturn724+ diffractometer	3073 reflections with *I* > 2σ(*I*)
Radiation source: Rotating Anode	*R*_int_ = 0.029
graphite	θ_max_ = 27.5°, θ_min_ = 3.3°
Detector resolution: 28.5714 pixels mm^-1^	*h* = −14→12
Multi–scan	*k* = −14→8
16790 measured reflections	*l* = −24→24
3165 independent reflections	

### Refinement

**Table:**

Refinement on *F*^2^	Primary atom site location: structure-invariant direct methods
Least-squares matrix: full	Secondary atom site location: difference Fourier map
*R*[*F*^2^ > 2σ(*F*^2^)] = 0.034	Hydrogen site location: inferred from neighbouring sites
*wR*(*F*^2^) = 0.085	H atoms treated by a mixture of independent and constrained refinement
*S* = 1.00	*w* = 1/[σ^2^(*F*_o_^2^) + (0.0519*P*)^2^ + 0.356*P*] where *P* = (*F*_o_^2^ + 2*F*_c_^2^)/3
3165 reflections	(Δ/σ)_max_ = 0.001
325 parameters	Δρ_max_ = 0.21 e Å^−^^3^
0 restraints	Δρ_min_ = −0.18 e Å^−^^3^

### Special details

**Table:**

Geometry. All e.s.d.'s (except the e.s.d. in the dihedral angle between two l.s. planes) are estimated using the full covariance matrix. The cell e.s.d.'s are taken into account individually in the estimation of e.s.d.'s in distances, angles and torsion angles; correlations between e.s.d.'s in cell parameters are only used when they are defined by crystal symmetry. An approximate (isotropic) treatment of cell e.s.d.'s is used for estimating e.s.d.'s involving l.s. planes.
Refinement. Refinement of *F*^2^ against ALL reflections. The weighted *R*-factor *wR* and goodness of fit *S* are based on *F*^2^, conventional *R*-factors *R* are based on *F*, with *F* set to zero for negative *F*^2^. The threshold expression of *F*^2^ > σ(*F*^2^) is used only for calculating *R*-factors(gt) *etc*. and is not relevant to the choice of reflections for refinement. *R*-factors based on *F*^2^ are statistically about twice as large as those based on *F*, and *R*- factors based on ALL data will be even larger.

### Fractional atomic coordinates and isotropic or equivalent isotropic displacement parameters (Å^2^)

**Table:**

	*x*	*y*	*z*	*U*_iso_*/*U*_eq_	
O1	0.38167 (12)	0.37232 (12)	0.65858 (7)	0.0230 (3)	
O2	0.41296 (12)	0.53784 (11)	0.46276 (6)	0.0199 (3)	
O3	0.34305 (13)	0.65688 (12)	0.37900 (7)	0.0278 (3)	
O4	0.10378 (13)	0.37757 (11)	0.70404 (7)	0.0232 (3)	
O5	0.31311 (13)	0.40633 (13)	0.76801 (7)	0.0239 (3)	
O6	0.53506 (12)	0.62167 (12)	0.58354 (7)	0.0230 (3)	
O7	0.60240 (13)	0.75251 (14)	0.50554 (8)	0.0299 (3)	
O8	0.13104 (11)	0.65130 (11)	0.71188 (7)	0.0185 (3)	
O9	0.05804 (12)	0.63399 (13)	0.82130 (7)	0.0266 (3)	
C1	0.29255 (16)	0.52505 (16)	0.48909 (9)	0.0175 (4)	
H1	0.2496	0.6001	0.4821	0.021*	
C2	0.23307 (18)	0.43133 (17)	0.44501 (10)	0.0232 (4)	
H2A	0.2322	0.4557	0.3952	0.028*	
H2B	0.2788	0.3582	0.4485	0.028*	
C3	0.10758 (18)	0.41011 (18)	0.46967 (10)	0.0232 (4)	
H3A	0.0705	0.3512	0.4389	0.028*	
H3B	0.0620	0.4831	0.4649	0.028*	
C4	0.10019 (17)	0.36823 (16)	0.54617 (9)	0.0199 (4)	
C5	0.17123 (17)	0.45659 (15)	0.59208 (9)	0.0170 (4)	
H5	0.1220	0.5287	0.5925	0.020*	
C6	0.18088 (17)	0.42097 (15)	0.66877 (9)	0.0170 (4)	
C7	0.30344 (17)	0.44315 (16)	0.69929 (9)	0.0193 (4)	
C8	0.33202 (17)	0.57276 (15)	0.69257 (9)	0.0174 (4)	
C9	0.32401 (16)	0.60840 (15)	0.61315 (9)	0.0168 (4)	
H9	0.2514	0.6575	0.6096	0.020*	
C10	0.29698 (16)	0.49793 (15)	0.56786 (9)	0.0169 (4)	
C11	0.42551 (17)	0.68795 (16)	0.59020 (10)	0.0205 (4)	
H11	0.4056	0.7232	0.5437	0.025*	
C12	0.44686 (19)	0.78558 (17)	0.64350 (10)	0.0242 (4)	
H12A	0.3891	0.8486	0.6350	0.029*	
H12B	0.5268	0.8180	0.6360	0.029*	
C13	0.43596 (18)	0.74467 (19)	0.72063 (10)	0.0249 (4)	
H13	0.4940	0.7854	0.7516	0.030*	
C14	0.45021 (17)	0.61168 (18)	0.72499 (9)	0.0228 (4)	
H14A	0.4585	0.5849	0.7741	0.027*	
H14B	0.5184	0.5842	0.6971	0.027*	
C15	0.24973 (16)	0.64528 (17)	0.73956 (10)	0.0189 (4)	
H15	0.2465	0.6081	0.7869	0.023*	
C16	0.31041 (19)	0.76122 (18)	0.74652 (10)	0.0247 (4)	
C17	0.2620 (2)	0.8570 (2)	0.77153 (13)	0.0365 (5)	
H17A	0.1823	0.8563	0.7870	0.044*	
H17B	0.3069	0.9266	0.7741	0.044*	
C18	−0.03029 (17)	0.37090 (18)	0.56763 (10)	0.0251 (4)	
H18A	−0.0771	0.3276	0.5332	0.030*	
H18B	−0.0577	0.4516	0.5694	0.030*	
H18C	−0.0394	0.3352	0.6140	0.030*	
C19	0.14350 (18)	0.24192 (16)	0.55248 (11)	0.0254 (4)	
H19A	0.0879	0.1901	0.5286	0.031*	
H19B	0.1486	0.2204	0.6021	0.031*	
H19C	0.2216	0.2349	0.5308	0.031*	
C20	0.38806 (17)	0.40401 (16)	0.58546 (9)	0.0197 (4)	
H20A	0.3734	0.3345	0.5561	0.024*	
H20B	0.4682	0.4332	0.5746	0.024*	
C21	0.42336 (18)	0.60148 (16)	0.40372 (9)	0.0215 (4)	
C22	0.54572 (18)	0.59762 (19)	0.37348 (10)	0.0269 (4)	
H22A	0.5877	0.6694	0.3854	0.032*	
H22B	0.5410	0.5900	0.3224	0.032*	
H22C	0.5884	0.5309	0.3929	0.032*	
C23	0.61675 (18)	0.66545 (18)	0.54004 (10)	0.0257 (4)	
C24	0.72644 (19)	0.5931 (2)	0.54079 (13)	0.0367 (5)	
H24A	0.7804	0.6225	0.5768	0.044*	
H24B	0.7649	0.5973	0.4948	0.044*	
H24C	0.7059	0.5122	0.5512	0.044*	
C25	0.04182 (17)	0.63722 (15)	0.75910 (10)	0.0196 (4)	
C26	−0.07351 (17)	0.62392 (18)	0.72259 (11)	0.0241 (4)	
H26A	−0.1368	0.6174	0.7574	0.029*	
H26B	−0.0719	0.5537	0.6935	0.029*	
H26C	−0.0877	0.6919	0.6928	0.029*	
H5O	0.247 (3)	0.370 (3)	0.7794 (16)	0.058 (10)*	

### Atomic displacement parameters (Å^2^)

**Table:**

	*U*^11^	*U*^22^	*U*^33^	*U*^12^	*U*^13^	*U*^23^
O1	0.0266 (7)	0.0260 (6)	0.0164 (6)	0.0104 (6)	0.0032 (5)	0.0041 (5)
O2	0.0214 (7)	0.0240 (6)	0.0143 (6)	−0.0021 (6)	0.0037 (5)	0.0021 (5)
O3	0.0346 (8)	0.0311 (7)	0.0178 (7)	−0.0017 (7)	−0.0006 (6)	0.0050 (6)
O4	0.0266 (7)	0.0249 (6)	0.0182 (6)	−0.0046 (6)	0.0036 (6)	0.0027 (5)
O5	0.0248 (8)	0.0322 (7)	0.0147 (7)	0.0030 (7)	−0.0002 (5)	0.0073 (5)
O6	0.0193 (7)	0.0290 (7)	0.0207 (6)	−0.0022 (6)	0.0025 (5)	0.0004 (6)
O7	0.0317 (8)	0.0329 (7)	0.0251 (7)	−0.0120 (7)	0.0042 (7)	0.0001 (6)
O8	0.0185 (7)	0.0225 (6)	0.0146 (6)	0.0010 (5)	−0.0010 (5)	0.0005 (5)
O9	0.0306 (8)	0.0325 (7)	0.0168 (6)	0.0021 (7)	0.0029 (6)	0.0005 (6)
C1	0.0188 (9)	0.0203 (8)	0.0133 (8)	0.0004 (7)	0.0029 (7)	0.0001 (7)
C2	0.0298 (11)	0.0267 (10)	0.0131 (8)	−0.0054 (9)	0.0009 (8)	−0.0024 (7)
C3	0.0263 (10)	0.0281 (9)	0.0151 (8)	−0.0050 (9)	−0.0009 (8)	−0.0017 (7)
C4	0.0219 (10)	0.0218 (8)	0.0160 (8)	−0.0032 (8)	0.0007 (7)	−0.0012 (7)
C5	0.0197 (9)	0.0180 (8)	0.0132 (8)	0.0023 (7)	0.0002 (7)	0.0003 (7)
C6	0.0212 (9)	0.0139 (7)	0.0160 (8)	0.0038 (7)	0.0018 (7)	0.0000 (6)
C7	0.0229 (10)	0.0231 (9)	0.0119 (8)	0.0040 (8)	0.0005 (7)	0.0029 (7)
C8	0.0177 (9)	0.0216 (8)	0.0130 (8)	0.0016 (7)	−0.0006 (7)	0.0014 (7)
C9	0.0173 (9)	0.0203 (8)	0.0129 (8)	0.0002 (7)	−0.0002 (7)	0.0003 (7)
C10	0.0195 (9)	0.0183 (8)	0.0130 (8)	0.0003 (7)	0.0000 (7)	−0.0006 (7)
C11	0.0216 (10)	0.0218 (9)	0.0181 (8)	−0.0011 (8)	0.0005 (7)	0.0012 (7)
C12	0.0259 (10)	0.0244 (9)	0.0223 (9)	−0.0056 (8)	0.0001 (8)	−0.0022 (8)
C13	0.0243 (10)	0.0326 (10)	0.0178 (9)	−0.0048 (9)	−0.0021 (8)	−0.0045 (8)
C14	0.0191 (10)	0.0339 (10)	0.0154 (8)	0.0001 (8)	−0.0042 (7)	−0.0001 (8)
C15	0.0176 (9)	0.0245 (9)	0.0147 (8)	0.0002 (8)	−0.0030 (7)	−0.0019 (7)
C16	0.0275 (11)	0.0291 (9)	0.0175 (9)	−0.0044 (9)	−0.0018 (8)	−0.0064 (8)
C17	0.0378 (13)	0.0326 (11)	0.0392 (13)	−0.0092 (10)	0.0053 (10)	−0.0127 (10)
C18	0.0232 (10)	0.0307 (10)	0.0214 (9)	−0.0046 (9)	−0.0002 (8)	−0.0016 (8)
C19	0.0314 (11)	0.0217 (9)	0.0232 (9)	−0.0052 (9)	0.0016 (8)	−0.0021 (8)
C20	0.0226 (10)	0.0207 (8)	0.0157 (8)	0.0033 (8)	0.0025 (7)	0.0022 (7)
C21	0.0299 (11)	0.0204 (9)	0.0142 (8)	−0.0039 (8)	0.0015 (7)	−0.0010 (7)
C22	0.0309 (11)	0.0297 (10)	0.0202 (9)	−0.0088 (9)	0.0070 (8)	−0.0012 (8)
C23	0.0241 (10)	0.0330 (10)	0.0199 (9)	−0.0097 (9)	0.0021 (8)	−0.0031 (8)
C24	0.0240 (11)	0.0475 (13)	0.0386 (13)	−0.0045 (10)	0.0078 (9)	−0.0033 (11)
C25	0.0236 (10)	0.0153 (8)	0.0199 (8)	0.0015 (7)	0.0040 (8)	0.0007 (7)
C26	0.0212 (10)	0.0246 (9)	0.0265 (10)	0.0005 (8)	0.0008 (8)	0.0006 (8)

### Geometric parameters (Å, °)

**Table:**

O1—C7	1.433 (2)	C10—C20	1.532 (3)
O1—C20	1.443 (2)	C11—C12	1.534 (3)
O2—C21	1.348 (2)	C11—H11	1.0000
O2—C1	1.461 (2)	C12—C13	1.549 (3)
O3—C21	1.207 (2)	C12—H12A	0.9900
O4—C6	1.210 (2)	C12—H12B	0.9900
O5—C7	1.381 (2)	C13—C16	1.518 (3)
O5—H5O	0.89 (3)	C13—C14	1.541 (3)
O6—C23	1.341 (2)	C13—H13	1.0000
O6—C11	1.462 (2)	C14—H14A	0.9900
O7—C23	1.209 (3)	C14—H14B	0.9900
O8—C25	1.364 (2)	C15—C16	1.507 (3)
O8—C15	1.446 (2)	C15—H15	1.0000
O9—C25	1.201 (2)	C16—C17	1.320 (3)
C1—C2	1.524 (3)	C17—H17A	0.9500
C1—C10	1.535 (2)	C17—H17B	0.9500
C1—H1	1.0000	C18—H18A	0.9800
C2—C3	1.517 (3)	C18—H18B	0.9800
C2—H2A	0.9900	C18—H18C	0.9800
C2—H2B	0.9900	C19—H19A	0.9800
C3—C4	1.538 (2)	C19—H19B	0.9800
C3—H3A	0.9900	C19—H19C	0.9800
C3—H3B	0.9900	C20—H20A	0.9900
C4—C18	1.534 (3)	C20—H20B	0.9900
C4—C19	1.539 (3)	C21—C22	1.502 (3)
C4—C5	1.565 (2)	C22—H22A	0.9800
C5—C6	1.522 (2)	C22—H22B	0.9800
C5—C10	1.572 (3)	C22—H22C	0.9800
C5—H5	1.0000	C23—C24	1.496 (3)
C6—C7	1.527 (3)	C24—H24A	0.9800
C7—C8	1.531 (3)	C24—H24B	0.9800
C8—C15	1.539 (3)	C24—H24C	0.9800
C8—C14	1.542 (3)	C25—C26	1.489 (3)
C8—C9	1.571 (2)	C26—H26A	0.9800
C9—C11	1.534 (3)	C26—H26B	0.9800
C9—C10	1.567 (2)	C26—H26C	0.9800
C9—H9	1.0000		
			
C7—O1—C20	114.25 (13)	C13—C12—H12B	108.9
C21—O2—C1	115.03 (14)	H12A—C12—H12B	107.8
C7—O5—H5O	108 (2)	C16—C13—C14	101.84 (16)
C23—O6—C11	116.35 (15)	C16—C13—C12	110.20 (17)
C25—O8—C15	116.29 (14)	C14—C13—C12	110.16 (16)
O2—C1—C2	107.14 (14)	C16—C13—H13	111.4
O2—C1—C10	109.03 (14)	C14—C13—H13	111.4
C2—C1—C10	114.22 (15)	C12—C13—H13	111.4
O2—C1—H1	108.8	C13—C14—C8	100.13 (15)
C2—C1—H1	108.8	C13—C14—H14A	111.7
C10—C1—H1	108.8	C8—C14—H14A	111.7
C3—C2—C1	110.94 (16)	C13—C14—H14B	111.7
C3—C2—H2A	109.5	C8—C14—H14B	111.7
C1—C2—H2A	109.5	H14A—C14—H14B	109.5
C3—C2—H2B	109.5	O8—C15—C16	114.46 (15)
C1—C2—H2B	109.5	O8—C15—C8	112.15 (14)
H2A—C2—H2B	108.0	C16—C15—C8	104.77 (15)
C2—C3—C4	113.27 (17)	O8—C15—H15	108.4
C2—C3—H3A	108.9	C16—C15—H15	108.4
C4—C3—H3A	108.9	C8—C15—H15	108.4
C2—C3—H3B	108.9	C17—C16—C15	125.55 (19)
C4—C3—H3B	108.9	C17—C16—C13	127.7 (2)
H3A—C3—H3B	107.7	C15—C16—C13	106.74 (16)
C18—C4—C3	107.40 (16)	C16—C17—H17A	120.0
C18—C4—C19	107.78 (16)	C16—C17—H17B	120.0
C3—C4—C19	110.65 (16)	H17A—C17—H17B	120.0
C18—C4—C5	109.48 (15)	C4—C18—H18A	109.5
C3—C4—C5	107.39 (15)	C4—C18—H18B	109.5
C19—C4—C5	113.95 (16)	H18A—C18—H18B	109.5
C6—C5—C4	113.54 (15)	C4—C18—H18C	109.5
C6—C5—C10	107.36 (15)	H18A—C18—H18C	109.5
C4—C5—C10	119.92 (14)	H18B—C18—H18C	109.5
C6—C5—H5	104.9	C4—C19—H19A	109.5
C4—C5—H5	104.9	C4—C19—H19B	109.5
C10—C5—H5	104.9	H19A—C19—H19B	109.5
O4—C6—C5	126.36 (18)	C4—C19—H19C	109.5
O4—C6—C7	120.91 (16)	H19A—C19—H19C	109.5
C5—C6—C7	112.72 (15)	H19B—C19—H19C	109.5
O5—C7—O1	106.88 (14)	O1—C20—C10	110.86 (14)
O5—C7—C6	112.48 (16)	O1—C20—H20A	109.5
O1—C7—C6	105.14 (14)	C10—C20—H20A	109.5
O5—C7—C8	111.17 (15)	O1—C20—H20B	109.5
O1—C7—C8	112.19 (15)	C10—C20—H20B	109.5
C6—C7—C8	108.86 (14)	H20A—C20—H20B	108.1
C7—C8—C15	110.56 (15)	O3—C21—O2	123.17 (18)
C7—C8—C14	115.67 (15)	O3—C21—C22	124.18 (17)
C15—C8—C14	97.78 (14)	O2—C21—C22	112.64 (17)
C7—C8—C9	108.82 (15)	C21—C22—H22A	109.5
C15—C8—C9	112.61 (15)	C21—C22—H22B	109.5
C14—C8—C9	111.15 (15)	H22A—C22—H22B	109.5
C11—C9—C10	118.26 (15)	C21—C22—H22C	109.5
C11—C9—C8	112.79 (15)	H22A—C22—H22C	109.5
C10—C9—C8	109.30 (14)	H22B—C22—H22C	109.5
C11—C9—H9	105.1	O7—C23—O6	123.7 (2)
C10—C9—H9	105.1	O7—C23—C24	125.3 (2)
C8—C9—H9	105.1	O6—C23—C24	111.01 (18)
C20—C10—C1	112.32 (14)	C23—C24—H24A	109.5
C20—C10—C9	108.66 (14)	C23—C24—H24B	109.5
C1—C10—C9	112.38 (14)	H24A—C24—H24B	109.5
C20—C10—C5	109.46 (14)	C23—C24—H24C	109.5
C1—C10—C5	108.62 (14)	H24A—C24—H24C	109.5
C9—C10—C5	105.13 (14)	H24B—C24—H24C	109.5
O6—C11—C9	110.50 (14)	O9—C25—O8	122.84 (17)
O6—C11—C12	107.79 (16)	O9—C25—C26	126.38 (17)
C9—C11—C12	111.49 (15)	O8—C25—C26	110.75 (15)
O6—C11—H11	109.0	C25—C26—H26A	109.5
C9—C11—H11	109.0	C25—C26—H26B	109.5
C12—C11—H11	109.0	H26A—C26—H26B	109.5
C11—C12—C13	113.19 (16)	C25—C26—H26C	109.5
C11—C12—H12A	108.9	H26A—C26—H26C	109.5
C13—C12—H12A	108.9	H26B—C26—H26C	109.5
C11—C12—H12B	108.9		
			
C21—O2—C1—C2	79.21 (18)	C8—C9—C10—C1	−177.79 (15)
C21—O2—C1—C10	−156.71 (14)	C11—C9—C10—C5	−164.89 (15)
O2—C1—C2—C3	178.82 (15)	C8—C9—C10—C5	64.24 (17)
C10—C1—C2—C3	58.0 (2)	C6—C5—C10—C20	52.48 (18)
C1—C2—C3—C4	−60.8 (2)	C4—C5—C10—C20	−79.04 (19)
C2—C3—C4—C18	170.35 (17)	C6—C5—C10—C1	175.44 (14)
C2—C3—C4—C19	−72.3 (2)	C4—C5—C10—C1	43.9 (2)
C2—C3—C4—C5	52.7 (2)	C6—C5—C10—C9	−64.08 (16)
C18—C4—C5—C6	68.7 (2)	C4—C5—C10—C9	164.41 (15)
C3—C4—C5—C6	−175.05 (16)	C23—O6—C11—C9	155.35 (15)
C19—C4—C5—C6	−52.1 (2)	C23—O6—C11—C12	−82.60 (19)
C18—C4—C5—C10	−162.57 (15)	C10—C9—C11—O6	−55.2 (2)
C3—C4—C5—C10	−46.3 (2)	C8—C9—C11—O6	74.07 (18)
C19—C4—C5—C10	76.7 (2)	C10—C9—C11—C12	−175.06 (15)
C4—C5—C6—O4	−40.0 (2)	C8—C9—C11—C12	−45.8 (2)
C10—C5—C6—O4	−174.96 (17)	O6—C11—C12—C13	−81.5 (2)
C4—C5—C6—C7	138.70 (16)	C9—C11—C12—C13	39.9 (2)
C10—C5—C6—C7	3.77 (19)	C11—C12—C13—C16	−91.6 (2)
C20—O1—C7—O5	−176.40 (15)	C11—C12—C13—C14	20.0 (2)
C20—O1—C7—C6	63.87 (18)	C16—C13—C14—C8	44.81 (17)
C20—O1—C7—C8	−54.3 (2)	C12—C13—C14—C8	−72.12 (19)
O4—C6—C7—O5	1.3 (2)	C7—C8—C14—C13	−169.77 (16)
C5—C6—C7—O5	−177.53 (14)	C15—C8—C14—C13	−52.46 (16)
O4—C6—C7—O1	117.22 (17)	C9—C8—C14—C13	65.50 (17)
C5—C6—C7—O1	−61.59 (18)	C25—O8—C15—C16	−104.33 (18)
O4—C6—C7—C8	−122.38 (18)	C25—O8—C15—C8	136.49 (15)
C5—C6—C7—C8	58.80 (19)	C7—C8—C15—O8	−73.12 (19)
O5—C7—C8—C15	−57.8 (2)	C14—C8—C15—O8	165.67 (15)
O1—C7—C8—C15	−177.47 (14)	C9—C8—C15—O8	48.8 (2)
C6—C7—C8—C15	66.59 (18)	C7—C8—C15—C16	162.17 (15)
O5—C7—C8—C14	52.1 (2)	C14—C8—C15—C16	40.96 (17)
O1—C7—C8—C14	−67.5 (2)	C9—C8—C15—C16	−75.87 (18)
C6—C7—C8—C14	176.51 (14)	O8—C15—C16—C17	43.7 (3)
O5—C7—C8—C9	177.99 (15)	C8—C15—C16—C17	167.0 (2)
O1—C7—C8—C9	58.37 (19)	O8—C15—C16—C13	−137.21 (16)
C6—C7—C8—C9	−57.57 (19)	C8—C15—C16—C13	−13.96 (19)
C7—C8—C9—C11	−137.05 (16)	C14—C13—C16—C17	160.0 (2)
C15—C8—C9—C11	100.02 (18)	C12—C13—C16—C17	−83.1 (3)
C14—C8—C9—C11	−8.5 (2)	C14—C13—C16—C15	−19.07 (19)
C7—C8—C9—C10	−3.3 (2)	C12—C13—C16—C15	97.83 (19)
C15—C8—C9—C10	−126.24 (16)	C7—O1—C20—C10	−6.8 (2)
C14—C8—C9—C10	125.19 (16)	C1—C10—C20—O1	−174.21 (15)
O2—C1—C10—C20	−46.06 (19)	C9—C10—C20—O1	60.82 (19)
C2—C1—C10—C20	73.7 (2)	C5—C10—C20—O1	−53.47 (19)
O2—C1—C10—C9	76.83 (18)	C1—O2—C21—O3	9.4 (2)
C2—C1—C10—C9	−163.36 (16)	C1—O2—C21—C22	−171.83 (15)
O2—C1—C10—C5	−167.28 (13)	C11—O6—C23—O7	−2.5 (3)
C2—C1—C10—C5	−47.5 (2)	C11—O6—C23—C24	177.06 (17)
C11—C9—C10—C20	78.01 (19)	C15—O8—C25—O9	8.8 (2)
C8—C9—C10—C20	−52.86 (19)	C15—O8—C25—C26	−169.77 (15)
C11—C9—C10—C1	−46.9 (2)		

### Hydrogen-bond geometry (Å, °)

**Table:**

*D*—H···*A*	*D*—H	H···*A*	*D*···*A*	*D*—H···*A*
O5—H5O···O3^i^	0.89 (3)	2.18 (3)	2.853 (2)	133 (3)

Symmetry codes: (i) −*x*+1/2, −*y*+1, *z*+1/2.
